# 3-Methylthiopropionic Acid of *Rhizoctonia solani* AG-3 and Its Role in the Pathogenicity of the Fungus

**DOI:** 10.5423/PPJ.OA.08.2015.0159

**Published:** 2016-04-01

**Authors:** Frederick Kankam, Hai-Tao Long, Jing He, Chun-hong Zhang, Hui-Xiu Zhang, Lumei Pu, Huizhen Qiu

**Affiliations:** 1 College of Resources and Environmental Sciences/Gansu Provincial Key Lab of Aridland Crop Science, Gansu Agricultural University, Lanzhou 730070, China; 2 College of Sciences, Gansu Agricultural University Lanzhou 730070, China; 3 University for Development Studies, Faculty of Agriculture, Tamale, Ghana

**Keywords:** cell membrane, 3-methylthiopropionic acid, phytotoxin, *Rhizoctonia solani*, stem canker

## Abstract

Studies were conducted to determine the role of 3-methylthioproprionic acid (MTPA) in the pathogenicity of potato stem canker, *Rhizoctonia solani*, and the concentrations required to inhibit growth of *R. solani* under laboratory and plant house-based conditions. The experiments were laid out in a completely randomized design with five treatments and five replications. The treatments were 0, 1, 2, 4, and 8 mM concentrations of MTPA. The purified toxin exhibited maximal activity at pH 2.5 and 30°C. MTPA at 1, 2, 4, and 8 mM levels reduced plant height, chlorophyll content, haulm fresh weight, number of stolons, canopy development, and tuber weight of potato plants, as compared to the control. MTPA significantly affected mycelial growth with 8 mM causing the highest infection. The potato seedlings treated with MTPA concentrations of 1.0–8.0 mM induced necrosis of up to 80% of root system area. Cankers were resulted from the injection of potato seedling stems with 8.0 mM MTPA. The results showed the disappearance of cell membrane, rough mitochondrial and cell walls, change of the shape of chloroplasts, and swollen endoplasmic reticulum. Seventy-six (76) hours after toxin treatment, cell contents were completely broken, cytoplasm dissolved, and more chromatin were seen in the nucleus. The results suggested that high levels of the toxin concentration caused cell membrane and cytoplasm fracture. The integrity of cellular structure was destroyed by the phytotoxin. The concentrations of the phytotoxin were significantly correlated with pathogenicity and caused damage to the cell membrane of potato stem base tissue.

*Rhizoctonia solani* Kühn (teleomorph Thanatephorus cucumeris [Frank Donk]), the causal agent of Rhizoctonia canker and black scurf of potato (*Solanum tuberosum* L.) decreases yield substantially in worldwide. Either or both diseases may occur in an individual potato plant (Banville et al., 1997). *R. solani* is capable of causing disease on tubers, stems and stolons at all stages of the potato plant (Naz et al., 2008; Simons and Gilligan, 1997). Symptoms on stems and stolons consist of dark brown, necrotic lesions (Tsror and Peretz-Alon, 2005). In severely infested fields, these symptoms can result in death of sprouts and stems and girdling of stolons, resulting in malformed tubers and reduced yields due to poor stand emergence (Atkinson et al., 2010). Before disease infection, *R. solani* comes into contact with the plant and the runner hyphae grow longitudinally along the sprout between the epidermal cell walls (Hofman and Jongebloed, 1988). After this initial contact, the original hyphae produce primary mycelial branches developing into secondary branches which branches several times to form infection cushions (Chand et al., 1985; Hofman and Jongebloed, 1988).

Several plant pathogenic fungi produce toxic metabolites which contribute to symptom development in the infested plant (Schafer, 1994; Yoder, 1980). These toxins are divided into the host-selective toxins, which are toxic to hosts of the producing pathogens and show little or no toxicity to non-susceptible plants; and the non-selective toxins, which are synthesized by several fungi and can damage a wide range of host plant including plant species that are not normally attacked by the pathogen in nature. An example of non-selective phytotoxin is fusicoccin, produced by *Fusicoccum amygdali*, which induces stomata opening by stimulating K+-uptake into the guard cells, leading to uncontrolled transpiration and wilting (Aducci et al., 1997). Cell wall degrading enzymes are also involved in the process of infection and the establishment of a parasitic fungal–plant interaction (Friesen et al., 2008). There are numerous reports of plant growth-regulating activity and phytotoxicity of 3-methylthioproprionic acid (MTPA) on various species of plants. Carboxylic acid, including tiglic, phenylacetic, isovaleric, 3-methylthiopropionic and trans-3-methylthioacrylic acid, isolated from liquid cultures of the rice pathogen, *Xanthomonas campestris* pv. *oryzae* (Noda et al., 1980), have been reported to possess phytotoxic activity against rice seedlings in the form of necrosis, chlorosis or wilting (Patil, 1974).

There have been several conflicting reports and methodological limitations on the specific role of MTPA in the development of Rhizoctonia disease symptoms and the concentrations required to induce host responses. Furthermore, limited information is available regarding the role of MTPA in plant growth and development or in Rhizoctonia disease. An understanding of the role of MTPA in the disease process would help in improving in vitro screening for *R. solani* resistance as well as developing new strategies in disease management strategies.

The objective of this study was to investigate the role of the MTPA in Rhizoctonia disease using isolates of *R. solani* AG-3 on potato and determine the concentration at which MTPA will induce disease symptoms on potato in the absence of the fungus.

## Materials and Methods

### Soil sterilization, source of seed, and MTPA

Sandy loam soil was collected from the surface of protected cultivation plots with a 6-year history of continuous potato cropping and high incidence and severity of stem canker disease, in Lanzhou, China. The soil was sterilized in an autoclave at a temperature of 121°C and a pressure of 1.02 kg/cm^3^ for 30 min. Seeds of potato cv. Leshu and MTPA were obtained from the Gansu Provincial Key Lab of Aridland Crop Science, Gansu Agricultural University, Lanzhou, China.

### Potato stem canker response to MTPA

Seven sprouted seeds of potato cv. Leshu were planted in plastic pots (10 cm diameter, 7-cm deep) containing a 1:1 mixture of pasteurized peat and sand. The pots were placed in a 22°C growth chamber illuminated by fluorescent bulbs emitting 450 μmol/m/s of photosynthetically active radiation set to a photoperiod of 14 h of light. The plants were watered as needed with distilled H_2_O for 2 weeks.

Aqueous solutions of MTPA were standardized to pH 2.5 and filter-sterilized. All solutions were prepared to a final total concentration of 0, 1, 2, 4 and 8.0 mM. Fifty microliters of each solution were injected into the base of the potato stems using a sterile 1 cc syringe and 26-gauge needle (Becton, Dickinson and Co., Franklin Lakes, New Jersey). Control plants were injected with distilled H_2_O without MTPA solution. Each treatment was replicated five times. The pots were arranged in a randomized complete block design in the plant house. The experimental plants were watered 24 h after injection, with di-H_2_O. After 90 days, the plants were harvested from the pots and measurements on root and shoot length, number of lateral roots, incidence of root necrosis, and length of stem canker for each plant were recorded. The experiment was conducted twice.

### Rhizoctonia growth response to MTPA

Following the method of Bartz (2010), potato dextrose (PD) broth medium was balanced to pH 2.5 (that was helpful for pathogenesis), and autoclaved at 121°C and at approximately 16 psig for 45 min, and then amended with a filter-sterilized aqueous solution of MTPA standardized to pH 2.5. Amended media were adjusted to various concentrations of MTPA: 1.0, 2.0, 4.0 and 8.0 mM for use. Isolate of *R. solani* AG-3 and MTPA concentration each in 200 ml media were dispensed into five replicate 500 ml Erlenmeyer flasks. Each flask was inoculated with one 6 mm diameter plug of mycelium from an actively growing culture of *R. solani* isolate. Five flasks containing non-amended PD broth without inoculation of *R. solani* culture served as controls. Flasks were arranged in a randomized complete block design and incubated in the dark at 25°C. After 3 weeks of incubation, the mycelium was separated from the broth onto pre-tared grade 103 qualitative filter papers (Millipore, Billerica, Massachusetts) using a vacuum pump. The mycelia and filter papers were placed in a drying oven at 60°C for 2 days and equilibrated to room temperature in a desiccator for 15 min before the mycelial dry weight measurements were taken. The experiment was conducted twice.

### Fungal culture filtrate and toxin production

Cultures of a virulent isolate of *R. solani* were grown on PDA plates at 25°C for 6 days. Several plugs (6 mm diameter) of actively growing mycelium on PDA were cut with a cork borer. One disc was aseptically transferred into a 250 ml flask containing 100 ml PSB. Twenty-four flasks were placed on a rotary shaker (180 rpm) and incubated at 25°C for 3 days and then stored at 4°C. For production of toxin, two mycelium groups from PDB cultures were transferred into a 500 ml flask containing 200 ml of Richard’s medium. *R. solani* produces toxins in this type of medium (Lu et al*.*, 2005). The cultures were then incubated under stationary conditions at 25°C on artificial vibration once a day for 18 days. Culture filtrates were obtained by passing the liquid through four layers of cheesecloth and Whatman No. 1 filter paper. The experiment was conducted twice.

### Bioassay for phytotoxicity

Fungal culture filtrates were tested on 2-week-old potato plants in the greenhouse. Potato tubers were green-sprouted and planted in Perlite in 20-cm plastic pots. Two weeks after planting, the toxic culture filtrate fraction was serially diluted. 8 mM of the concentration was added to the individual pots. All tests consisted of four dilutions and five pots per dilution. After 8 weeks, foliar symptoms and plant height were recorded, and the plants uprooted from the Perlite. The roots were rinsed with tap water and visually rated. All resultant root damages were recorded on a scale of 1–5 (1- no damage and 5- complete root tip necrosis). The treated plants were compared with the control plants.

To allow more environmental control and to facilitate the handling of larger numbers of potato clones, a bioassay for potatoes was developed. Seeds were green sprouted, and the individual sprouts were removed from the whole potato (a 2-cm cube was removed with a scalpel). The cubes containing the green sprouts were grown in flats of Perlite for 1 week. At this time, the cubes were carefully removed from the Perlite, and the roots were rinsed with distilled water. The cubes were trimmed with a scalpel so that they could be individually lowered into 50-mI test tubes. The rooted sprout cubes were soaked for 5 min in a water solution containing 50 ml Dexon (*p*-dimethylaminobenzenediazo sodium sulfonate) and 50 ml benomyl/l of water to help minimize contamination. Toothpicks were inserted horizontally through the cubes to provide a suspended support over the mouths of the test tubes. Fifty millilitres of Hoagland’s solution containing streptomycin sulfate (50 mg/l) and chlortetracycline (50 mg/l) were added to each test tube. The root system from a sprouted cube was carefully lowered into the test tube, along with the cube, until the toothpick support rested on the rim of the tube. A black plastic sheath was placed around each tube to prevent illumination of the roots during the test period. The tubes were placed in a growth chamber (22°C, 5000 ft-c light intensity, 60% relative humidity for 3 days, with Hoagland’s solution added as necessary. After 3 days, any plant exhibiting abnormal signs of growth or non-uniformity in comparison with most plants in the chamber was discarded. Serially diluted toxin was added to the test tubes at this time (10 ml/tube, five tubes per dilution, three dilutions), and the plants were returned to the growth chamber for an additional 24–72 h. At this time, plants were removed from the test tubes, and root systems were rated on the 1–5 scale previously described.

### Influence of pH on toxin activity

To test the sensitivity of the toxin to pH, culture filtrates were adjusted to different pH levels of 2.0, 7.0 and 12.0 using 12 mol/l hydrochloric acid and 1 mol/l potassium hydroxide; incubated for 30 min and the pH was then adjusted to various levels and used. Each treatment was replicated three times. Sterile distilled water and the untreated culture filtrate served as controls.

### Disease assessment

At harvest, stem canker and stolons were evaluated on a scale of 0–5, as described by Tsror and Peretz-Alon (2005) where 0 = healthy tissue, 1 = several brown to black lesions, 2 = up to 15% of the tissue is covered with lesions, 3 = up to 30% of the tissue is covered with lesions, 4 = up to 60% of the tissue is covered with lesions and 5 = > 60% of the tissue is covered with lesions.

### Experimental design

The pots were arranged in a completely randomized design (CRD) comprising of 5 treatments, each replicated 5 times. The treatments consisted of four levels of MTPA (1, 2, 4 and 8 mM) and a control (0 mM).

### Data collection and analysis

Five plants were randomly sampled from the pots at 90 days after planting (DAP) to determine development of vegetative growth in potato plants. The roots of plants were washed with tap water. Data were collected on the following parameters: plant height, chlorophyll content of leaves (using SPAD apparatus), mycelial dry weight, root necrosis, root system, haulm fresh weight, number of stolons, number of symptomless stolons, canopy development, tuber weight, and number of tuber per plant, and stem canker. All data were subjected to Analysis of Variance Genstat (Version 11), and treatment means were separated using the least significant difference (LSD) at *P* < 0.05.

## Results

### The effect of pH on toxin activity

**Fig. 1:**
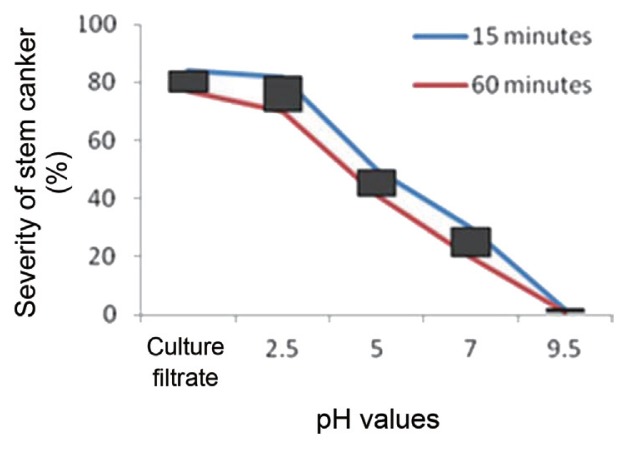
Effect of pH levels on toxin activities. The culture filtrates were held at the indicated pH for 15 or 60 minutes.

The influence of pH levels on phytotoxic activity in the culture filtrates of *R. solani* AG-3, presented in Fig. 1, indicate that maximal activity was recorded at pH 2.5, above which rapid decline occurred and complete inactivation was recorded at pH 9.5. The results demonstrate that a pH of 2.5 was helpful for the pathogenesis process of *R. solani*. The effectiveness of pH levels on toxin activities decreases as the pH increases (Fig. 1).

### Chlorophyll content

**Fig. 2:**
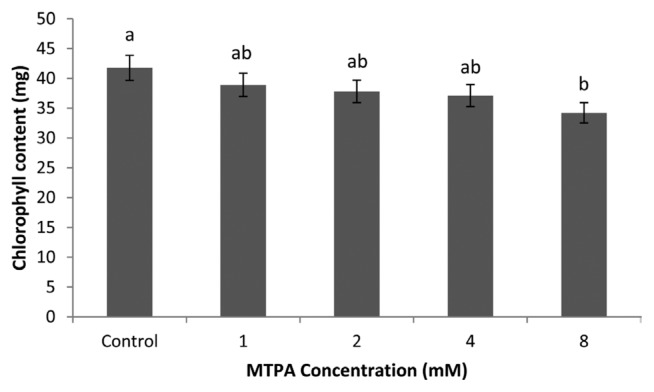
Effect of MTPA on chlorophyll content of leaves. Bars represent the standard error of the mean chlorophyll content for each treatment. Lengths with different letters are significantly different at *P* < 0.05.

No differences occurred between the control and MTPA 1, 2, 4 mM treatment regarding chlorophyll content, although the control pots had the highest chlorophyll content (Fig. 2). However, there were significant differences between 8 mM MTPA and the control.

### Potato seedling root response to MTPA

**Fig. 3:**
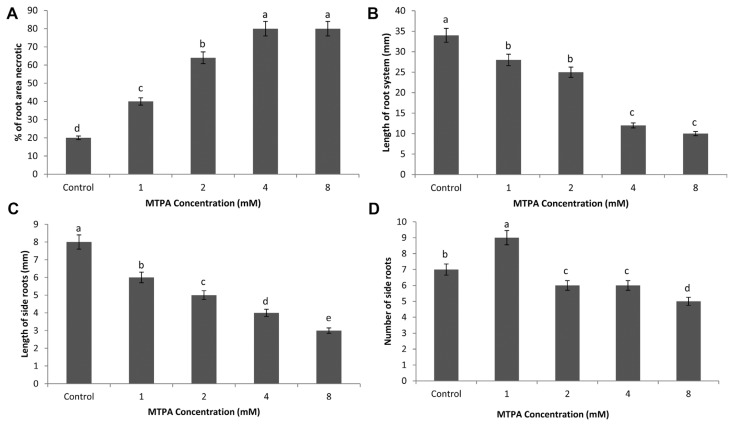
Effect of MTPA on physical plant parameters of potato plants grown from mini-tubers in soil mix. Bars represent the standard error of the mean response for each treatment. (A) Mean percent area of necrosis in root per plant. (B) Mean total root system length per plant. (C) Mean total length of lateral roots per plant. (D) Mean number of lateral roots per plant.

Root necrosis was significantly influenced by the MTPA application. Plants treated with 8 mM of MTPA phytotoxin produced significantly greater (*P* < 0.05) necrosis followed by 4, 2, 1 and 0 mM concentrations (Fig. 3A).

The concentration effect of MTPA on total potato seedling root length is shown in Fig. 3B. The mean root length of potato seedlings varied significantly between the various MTPA concentrations (*P* < 0.05). Root length of test plants were significantly reduced compared to control plants at the lowest concentration tested (1 mM) for MTPA and remained significantly less than the control for all increasing concentrations tested.

The effect of MTPA on lateral root system of potato length is shown in Fig. 3C. The mean lateral root system length varied significantly between seedlings treated with each concentration (*P* < 0.05). Plants had significantly shorter lateral roots compared to control plants when treated with MTPA 1, 2, 4 and 8 mM, respectively.

Fig. 3D shows the effect of MTPA on the number of lateral roots per potato seedling. The mean number of lateral roots varied significantly between seedlings treated with each concentration (*P* > 0.05). MTPA at 1 mM concentration significantly produced more lateral roots than the control plants (*P* < 0.05). At higher MTPA concentrations, plants produced fewer lateral roots, with a significant reduction at 8 mM (*P* < 0.05).

### Effect of MTPA on mycelial dry weight of *R. solani*

**Fig. 4:**
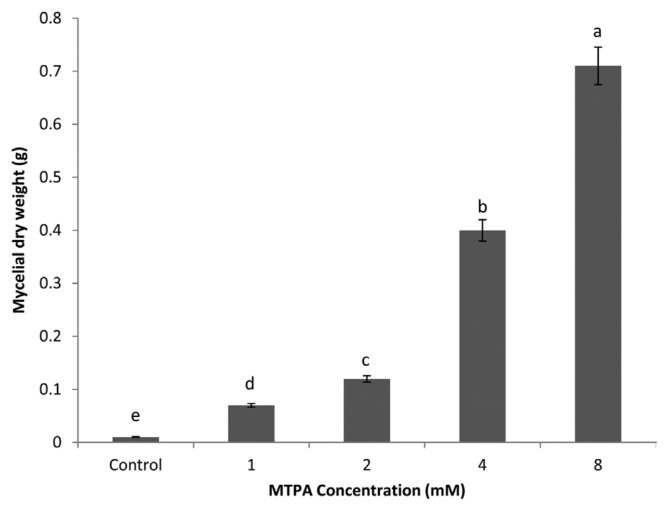
Effect of MTPA on mycelial dry weight of *Rhizoctonia solani*.

MTPA significantly increased (*P* < 0.05) the mycelial dry weight of *R. solani*. Generally, mycelial growth of the fungus increased as the concentration of the phytotoxins increased (Fig. 4). Plants treated with 8 mM of MTPA produced the heaviest mycelial dry weight followed by 4, 2, 0 mM/pot (Fig. 4).

### Plant height, haulm weight, number of stolons, and number of symptomless stolons

Table 1 shows the effect of MTPA on vegetative growth characteristics of potato plants at 75 days after planting. Generally, MTPA at 8 mM decreased plant height, haulm fresh weight, stolons number and stolons with no symtoms per plant, as compared with the control.

### Canopy development

Significant differences (*P* < 0.05) in canopy development (measured as percentage ground cover) were observed between plants treated with MTPA and the control (Table 2). Canopy development was generally lower in 8 mM MTPA treated pots.

### Assessment of yields and disease of potato plants

The effect of the phytotoxin on weight and number of tubers is shown in Table 3. The uninoculated plants produced more tubers by number and weight than the MTPA treatments. 8 mM MTPA treated plants produced significantly less (*P* < 0.05) tubers and lower yield by weight, compared to all other treatments.

Plants treated with 8 mM MTPA produced plants with the highest diseased stolons and stem canker indices (Table 3). This was followed by 4, 2, and 1 mM MTPA respectively. There was significant difference between 8 mM MTPA and the control (*P* < 0.05).

### Rhizoctonia stem canker and black scurf

**Fig. 5:**
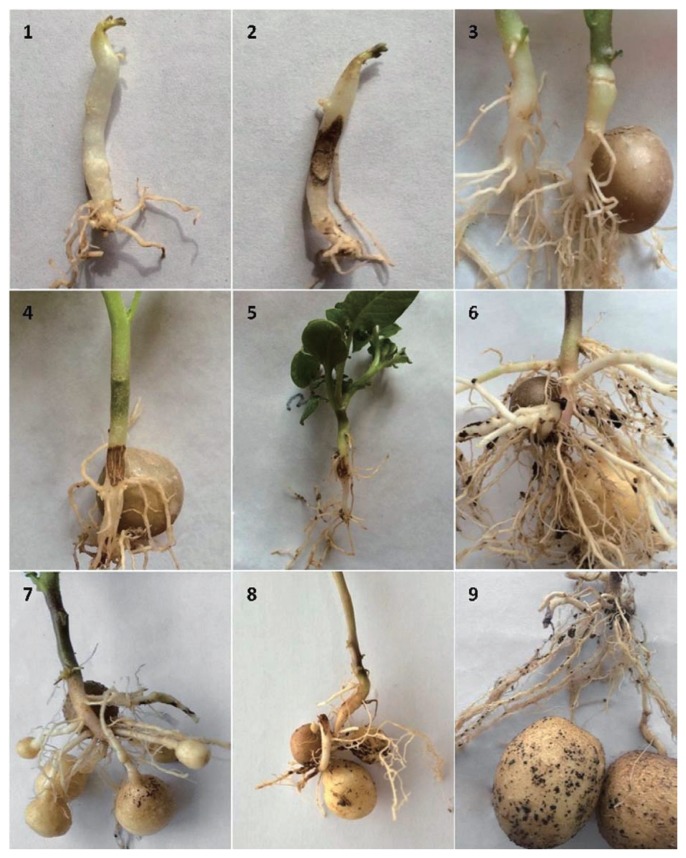
Progression of *Rhizoctonia solani* disease of potato. 1. Control potato buds after seeding 20 d, 2. Toxin treated potato buds show scab after seeding 20 d, 3. Control potato stems after seeding 35 d, 4. Treated potato stems show scab after seeding 35 d, 5. Treated potato roots show brown ulceration after seeding 40 d, 6. Treated potato stolons show scab after seeding 50 d, 7. Treated potato tubers show little black scurf after seeding 75 d, 8. Treatment potato tubers show little black scurf after seeding 90 d, 9. Treatment potato tubers show more black scurf after seeding 90 d.

Fig. 5 shows symptom progression of *R. solani* disease in toxin treated plants. Disease development starts with stolons, roots and stems showing brown lesions. As the lesions mature, they become cankers which eventually lead to necrosis. Between 75 to 90 days after seeding, the fungus forms dark brown to black, hard masses on the surface of the tuber which is referred to as black scurf.

### Effects of *R. solani* phytotoxin on tissue and cell

**Fig. 6:**
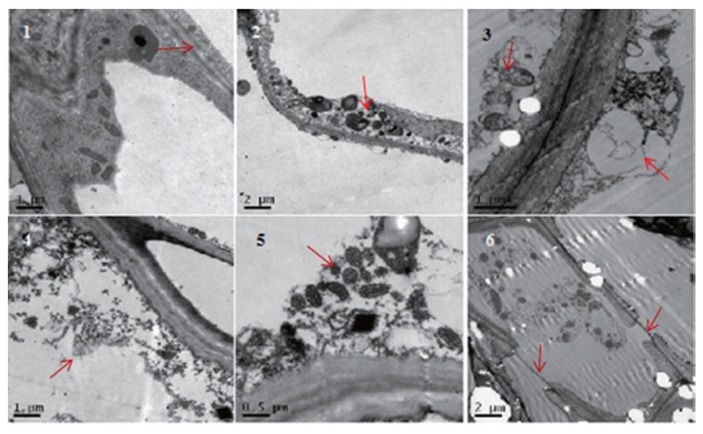
Destructive effects of *Rhizoctonia solani* phytotoxin on tissue and cell of potato. 1. The normal structure of cell membrane in control potato (×10,000), 2. Plasmids swelling 24 h after treatment (×5,000), 3. Endoplasmic reticulum swelling 48 h after treatment (×10,000), 4. Cell inclusions disappeared 72 h after treatment (×10,000), 5. Deformation of mitochondrial in treatment potato (×20,000), 6. Rupture of plasma membrane in treatment potato (×5,000).

The results showed that the cell membrane disappeared, mitochondria and cell wall was no longer smooth, shapes of chloroplast changed and endoplasmic reticulum swelled. Seventy-six (76) hours after treatment, cell contents were completely broken and eliminated, cytoplasm dissolved, and more chromatin can be seen in the nucleus (Fig. 6).

## Discussion

The implication of 3-methylthiopropionic acid (MTPA) in the infection process of Rhizoctonia on plants has led to a number of studies into the production of MTPA by isolates of *R. solani* from various AGs on a range of host plant species. There have been many reports of the effects of MTPA on plant growth, but few studies have directly linked MTPA to the development of symptoms typical of Rhizoctonia infection. Furthermore, reports that MTPA inhibits the growth of *R. solani* conflict with reports that the fungus produces and metabolizes MTPA to other chemically related compounds. Therefore, this study was conducted to determine the concentrations of MTPA required to induced root necrosis and stem cankers of potato in the absence of the fungus, the concentration at which MTPA inhibits the growth *R. solani* and the relationship between MTPA and production and disease symptom development on potato.

Incubation of toxin preparations at different pH values for either 15 or 60 min demonstrated that the toxin is more stable at pH range of 2.5. Hamdy (2008) found that purified collagenase from *R. solani* showed complete stability in the pH range of 4–5 and reasonable stability in the pH range of 3–6.5 after 1 h of exposure. The results of the study agree with Liang and Zheng (2012) who reported that the pH 2.0 was optimum for the phytotoxin of sheath-blight disease of rice caused by *R. solani*. The activity of MTPA was relatively stable in the range of pH levels 3.5–7.0 and decreased significantly beyond these limits. The minimum activity was observed above pH 7.0 contrary to Bartz et al. (2012) who observed that the optimal pH for the activity of phenylacetic acid (PAA) from *R. solani* infected tomato plant was 5.5. This suggests that the activity of MTPA may be due to their acidic nature. The relationship between pH and stem canker inhibition is the higher uptake of compounds by the seedlings at lower pH. Therefore, it can be concluded that the most plausible explanation for the observed phytotoxicity of the MTPA could be due to the elevated hydrogen ion activity of the MTPA concentrates.

The role of chlorophyll in plant is to take energy from the sun and assimilate CO_2_ (Suzuki et al., 1997). This indicates positive relationship between chlorophyll content and photosynthesis of plants (Eggink et al., 2001). Higher MTPA concentrations decreased chlorophyll content of plants as the luminous energy and electron transfer efficiency of MTPA treated plants were weaker than those of control treatments indirectly. This finding corroborates the work of Melis (2009) who observed that large amount of chlorophyll in plants collects more solar energy.

MTPA was found to damage potato seedlings by causing symptoms, typical to Rhizoctonia infection. The findings agree with Noda et al. (1980) and Perreaux et al. (1982) who reported leaf necrosis in rice and cassava respectively under MTPA treatment. Also, Robeson and Cook (1985) reported of protoplast collapse in cabbage dipped in 4.23 mM MTPA solutions. Contrary to these, Iacobellis and Devay (1987) identified no necrosis when bean hypocotyls and tobacco leaves were injected with 0.1–1 mM solutions of MTPA. A reduction in root length of MTPA treatment confirms the inhibitory effects of MTPA on the growth of potato as well as cassava (Iacobellis and Devay, 1987; Perreaux et al., 1982). Although a reduction in lateral root formation was observed on 8.0 mM MTPA solutions, Robeson and Cook (1985) reported of an increased adventitious root on cabbage stems injected with 1–4.2 mM concentrations of MTPA.

This study has identified differences in the effect of MTPA on mycelial yield. It was demonstrated that phytotoxins are essential for mycelial production, as very few or no mycelia were formed on the control treatments compared to greater mycelial dry weight on plants treated with MTPA. Differences in mycelial yields were also apparent between the MTPA concentrations, with 8 mM MTPA producing significantly greater mycelial yields than 4, 2 and 1 mM MTPA, respectively. Vegetative mycelial growth is known to precede sclerotial initiation and it is during this growth phase that any available nutrients in the substrate are absorbed by the fungus (Willets and Bullock, 1992).

Plants treated with 8 mM MTPA produced the shortest and poorly developed canopies compared to the other treatments and the control. The amendment of the soil with MTPA also reduced haulm fresh weight, tuber weight and number of tubers per plant. The poor performance of MTPA treatment with respect to growth and yield could be due to the pathogenic effect of *R. solani*, producing symptoms of stem canker on underground stems, stolons and tuber-borne sclerotia (Kyritsis and Wale, 2002). The higher incidence of diseased stolons and stem canker in MTPA treated plants could be due to the production of toxic metabolites, as reported by Robeson and Cook (1985). It could also be attributed to the effect of the toxins which induced pathogenicity in the potato plant, which resulted in chlorotic lesions and stem canker (Aducci et al., 1997).

Potato plants treated with *R. solani* phytotoxin resulted in Rhizoctonia disease at all growing stages which agrees Ahvenniemi et al. (2007) that Rhizoctonia disease develops in four phases. The first and second phases result in infection of stolon tips prior to and after seeding, respectively, whilst the 3rd and 4th phases result in scaby and black scurf development on progenies respectively (Ahvenniemi et al., 2007; Woodhall et al., 2008). Infected stolons resulted in death of sprouts and stems, malformed tubers and the formation of aerial tubers (Atkinson et al., 2010).

The results of the study suggested that high levels of toxin concentration caused cell membrane and cytoplasm fracture corroborating, Friesen et al. (2008) findings that fungi pathogens use toxins to elicit plant cell death and derive nutrition from the dead tissue. They further observed that phytotoxins may interact with a range of cellular targets, undermine membrane integrity, thereby disrupting the biosynthesis of crucial metabolites.

From the results obtained, it was observed that all the MTPA concentrations reduced the plant height, chlorophyll content of leaves, root system, haulm fresh weight, number of stolons, number of symptomless stolons, canopy development, tuber weight, and number of tuber per plant. Increasing the MTPA concentration resulted in corresponding increase in number of diseased stolons, mycelial dry weight, root necrosis and stem canker. The reductions in growth parameters and stem canker indices were found to be proportional to the MTPA level. It is therefore concluded that MTPA phytotoxin is involved in the pathogenicity of Rhizoctonia disease of potato at all concentrations and the damage is most severe at 8 mM MTPA. Furthermore, the integrity of cellular structure was destroyed by the phytotoxin. The concentrations of phytotoxin significantly correlated with pathogenicity and caused damage to cell membrane on potato stem base tissue.

## Figures and Tables

**Table 1:** Effect of MTPA on plant height, haulm fresh weight, number of stolons per plant, and number of symptomless stolons of potato plants

Treatment	Plant height (cm)	Haulm fresh weight (g)	No. of stolons/plant	No. of symptomless stolons
Control	40.10a	104.80a	4.00a	3.80a
1 mM	29.00b	91.30b	3.60ab	3.00ab
2 mM	23.20c	63.60c	3.00bc	2.20b
4 mM	18.90d	40.00d	2.10c	1.20c
8 mM	13.40e	29.10e	1.00d	0.10d
LSD (0.05)	2.67	1.66	0.94	0.83
CV (%)	5.70	1.30	18.30	21.40

Means followed by the same letters in a column are not significantly different (*P* > 0.05).

**Table 2:** Canopy development measured as percentage ground cover of plants

Treatment	% cover at 28 DAP	% cover at 35 DAP	% cover at 42 DAP	% cover at 49 DAP
Control	27.00a	43.00a	75.00a	92.30a
1 mM	23.73a	37.20b	66.70b	87.00b
2 mM	14.04b	30.40c	60.40c	75.00c
4 mM	13.41b	22.70d	49.30d	74.00c
8 mM	9.41b	12.00e	28.60e	65.00d
LSD (0.05)	5.41	3.44	3.08	1.85
s.e.d	2.34	1.49	1.34	0.80
CV (%)	15.30	6.30	2.90	1.20

Means followed by the same letters in a column are not significantly different (*P* > 0.05).

**Table 3:** Effect of MTPA on tuber production, diseased stolon and stem canker (0–5)

Treatment	Total tuber weight (g) per plant	Number of tubers per plant	Diseased stolons	% Stem canker
Control	27.83a	3.63a	2.00d	0.10b
1 mM	20.26b	2.40b	23.30c	0.75b
2 mM	17.64bc	1.80c	60.70b	3.00a
4 mM	15.00cd	1.71c	82.00a	3.00a
8 mM	13.90d	1.40c	90.00a	3.43a
LSD (0.05)	2.76	0.33	8.29	0.80
s.e.d	1.20	0.10	3.59	0.35
CV (%)	7.70	8.00	8.50	25.39

Numbers within the same column which share the same letters are not significantly different at *P* < 0.05.
